# A Multimodal Protocol Combining ^99m^Tc-Tilmanocept with Indocyanine Green Fluorescence Lympho-Angiography for Sentinel Lymph Node Biopsy in Early-Stage Oral Cancer: A Case Series

**DOI:** 10.3390/diagnostics14161805

**Published:** 2024-08-19

**Authors:** Andrea Galli, Carla Canevari, Emilio Salerno, Ayhan Irem, Marco Familiari, Carlo Pettirossi, Rosa Alessia Battista, Arturo Chiti, Mario Bussi, Leone Giordano

**Affiliations:** 1 Department of Otolaryngology-Head and Neck Surgery, IRCCS San Raffaele, Via Olgettina 60, 20132 Milan, Italy; salerno.emilio@hsr.it (E.S.);; 2 Faculty of Medicine, Vita-Salute San Raffaele University, Via Olgettina 58, 20132 Milan, Italy; 3 Unit of Nuclear Medicine, IRCCS San Raffaele, Via Olgettina 60, 20132 Milan, Italy; canevari.carla@hsr.it; 4 ASST Nord Milano, 20099 Milan, Italy

**Keywords:** tilmanocept, indocyanine green, sentinel lymph node biopsy, early stage, oral cancer

## Abstract

Sentinel lymph node biopsy (SLNB) is currently considered as a viable alternative to elective neck dissection (END) for the management of cN0 oral cavity squamous cell carcinoma (OCSCC). However, some difficulties were detected in sentinel lymph node (SLN) identification in floor of mouth (FOM) and ventral tongue tumors because of the so-called “shine-through radioactivity” of the injection site, which may mask nodal hotspots in proximity. We assessed the feasibility and the potential strengths of combining ^99m^Tc-Tilmanocept with indocyanine green (ICG) fluorescence lympho-angiography in a dedicated multimodal protocol for SLNB in T1/T2N0 oral cancer to evaluate the synergistic role of each of these two tracers in providing the appropriate sensitivity and ease of learning, even in such a critical anatomical subsite. A detailed, stepwise description of our multimodal protocol is provided, together with the presentation of its application in two cases of early-stage ventral tongue tumors. Radioactive guidance with ^99m^Tc-Tilmanocept was used preoperatively to perform planar lymphoscintigraphy and single-photon emission computed tomography/computed tomography and to define the nodal hotspot(s) and the surgical “roadmap”. In addition, it was used intraoperatively to pinpoint the SLN location within each nodal hotspot with high specificity but limited spatial resolution. Optical guidance with ICG injection at the tumor bed and near-infrared fluorescence imaging was then added, providing intuitive intraoperative guidance within each nodal hotspot with high spatial resolution. Our small experience with this protocol is illustrated and future perspectives are highlighted.

## 1. Introduction

Sentinel lymph node biopsy (SLNB) is currently considered as a viable alternative to elective neck dissection (END) for the management of cN0 oral cavity squamous cell carcinoma (OCSCC), reducing the morbidity of unnecessary END (about 60–70%) and enhancing the detection of occult micrometastasis and abnormal lymphatic drainages [1,2]. Nevertheless, some difficulties were pointed out in sentinel lymph node (SLN) identification in floor-of-mouth (FOM) and ventral tongue tumors as a result of the so-called “shine-through radioactivity” of the injection site, which may mask nodal hotspots in close proximity (level IB/IIA) [2,3]. Moreover, the inevitable learning curve of the procedure could represent an obstacle to its widespread adoption [1].

How can these limitations be overcome? One possible solution may be represented by systematic superselective level IB dissection: with this approach, the fat pad located between the submandibular gland (posteriorly), the lower edge of the mandible (cranially), the anterior belly of the digastric muscle (anteriorly), and the mylohyoid muscle (deeply) is regularly dissected during SLNB procedures regardless of lymphoscintigraphy-SPECT/CT findings [4]. In two prospective single-arm studies, this approach proved extremely reliable: Stoeckli et al. showed that 50% of the SLNs found in level I were identified only during surgical dissection of the “preglandular fat pad” (with negative preoperative lymphoscintigraphy-SPECT/CT) [4]; in Christensen et al.’s study, in 12 pts (24%), the neck level I was without a detectable SLN on preoperative SPECT/CT, but intraoperatively, a SLN could be detected optically in this neck level (systematically dissected) anterior to the submandibular gland [5]. Superselective level IB dissection is, thus, an effective strategy, although it may jeopardize the theoretical advantages of the SLNB procedure by increasing surgical time and skin incisions, placing the mandibular branch of the facial nerve at greater risk, especially if a completion neck dissection is required afterward due to positive SLN.

The introduction of ^99m^Tc-Tilmanocept as radiotracer for SLNB has provided a more specific tool for SLN identification in early-stage OCSCC because of its receptorial nature [6]. However, in a prospective within-patient assessment, den Toom et al. proved that the SLN-to-injection-site ratio was similar for both ^99m^Tc-Tilmanocept and the traditionally employed ^99m^Tc-nanocolloid (0.066, IQR 0.1 vs. 0.054, IQR 0.07; *p* = 0.232) because of a lower radioactive uptake in SLN for ^99m^Tc-Tilmanocept when compared to colloids (1.95% vs. 3.16%; *p* = 0.010); this underplays the potential advantages of this new radiotracer per se [7].

Optical guidance may aid in the intraoperative detection of SLN, although evidence regarding blue dye (e.g., methylene blue) has been inconsistent [1]. Indocyanine green (ICG) was more recently introduced as an alternative optical tracer, with the potential advantages of not staining the injection site under white light and being detectable through overlying soft tissue (usually to a maximum of 1–2 cm), as opposed to blue dye [8,9]. Nevertheless, both fluorescent and blue dye drain quickly and passively to SLN without being retained within them, with a rapid washout and, therefore, uptake in downstream non-SLN (i.e., unrestricted lymphatic migratory pattern). This makes their washout likely at the time of nodal dissection, especially when dealing with multiple SLNs. Therefore, it is advised not to perform SLNB in the head and neck region with an optical tracer alone [1]. A possible solution may be provided by the coupling of ICG to a colloid; this scenario was assessed in a recent Danish series (Christensen A et al., 2023) [5] in which a hybrid, bimodal tracer was created through non-covalent binding between ICG and ^99m^Tc-nanocolloid and used in 50 patients affected by tumors located in the anterior oral cavity (i.e., FOM, inferior surface of tongue, buccal mucosa, and lower gum). The false omission rate for this protocol was 0%, and in 46% of the patients, additional SLNs in level I were detected only with optical guidance; however, SLN in the “preglandular fat pad” of level IB was still identified only during superselective dissection together with fluorescence imaging, since the hotspot was masked on preoperative lymphoscintigraphy and SPECT/CT due to the “shine-through phenomenon” [5]. 

Conversely, the combination of ^99m^Tc-Tilmanocept with ICG in a single, hybrid tracer is not yet approved for clinical use, although it could overcome the need for a systematic superselective level IB dissection from a theoretical standpoint. Indeed, the residual radioactivity in the injection site is significantly lower when compared to colloids (29.9% vs. 60.9%; *p* < 0.001), underplaying the role of “shine-through radioactivity” per se. Therefore, our idea was to combine ^99m^Tc-Tilmanocept with ICG in a multimodal protocol to enhance the potential advantages of each of these two modern tracers, aiming at providing the appropriate sensitivity and ease of learning for the adoption of SLNB in T1/T2N0 oral cancer on a wider scale, even for the more critical anatomical subsites such as the ventral tongue and the FOM. Accordingly, we applied the proposed protocol in a few cases to test its feasibility.

## 2. Operative Technique

### 2.1. Case #1

**Figure 1:**
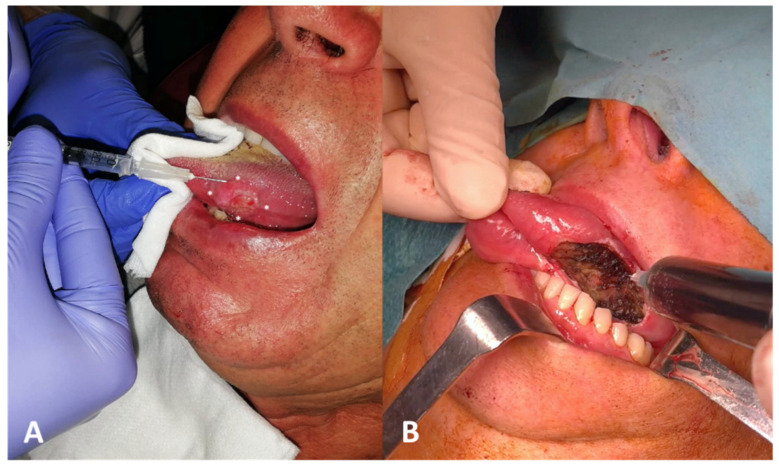
(**A**) Preoperative ^99m^Tc-Tilmanocept submucosal injections around the tumor at cardinal points (asterisks) in healthy mucosa. (**B**) Indocyanine green injection directly at the margins of the tumor bed with four peripheral injections immediately after transoral laser excision.

**Figure 2:**
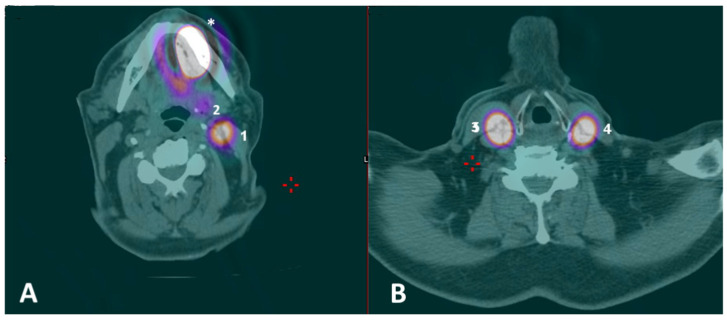
(**A**,**B**) Preoperative SPECT/CT with identification of multiple nodal hotspots (lighter circular areas encircled by a purplish ring, or, for (2), the purplish region) at levels IB (2), IIA (1) and III (4) on the left side and at level III (3) on the right side; hotspot at the primary injection site was also visible (asterisk).

A 71-year-old man with a histologically proven left ventral tongue margin cT1 cN0 squamous cell carcinoma (SCC) was submitted to this protocol after a thorough radiological assessment (head and neck contrast-enhanced magnetic resonance imaging, neck ultrasound) had excluded obvious lymph node metastasis (Video S1). A preoperative lymphoscintigraphy with ^99m^Tc-Tilmanocept (Lymphoseek^®^, Navidea Biopharmaceuticals, Inc.; Dublin, OH, USA) was performed about 4 h before surgery, with four submucosal injections around the tumor using an insulin syringe with a 27-gauge needle (0.1 mL per injection, 0.4 mL of total dose, 18 MBq) at cardinal points in healthy mucosa (Figure 1). Immediately after radioisotope injection, planar lymphoscintigraphic images were obtained using a GE Millennium gamma camera, with a high-resolution collimator. A support under the shoulders was positioned to reproduce operative conditions. The acquisition window was set at 140 Kev (±10%); the planar image matrix was 256 × 256. The images were obtained in anterior and posterior views. In addition, we performed single-photon emission computed tomography/computed tomography (SPECT/CT) to provide tomographic acquisition and improve anatomical SLN location. Dynamic acquisition allowed differentiation of the first lymph node relays (proper SLN(s)) from secondary ones: in this case, nodal hotspots were identified at levels IB, IIA and III on the left side and at level III on the right side (Figure 2). Skin landmarking was performed at the end of procedure to aid surgical planning.

**Figure 3:**
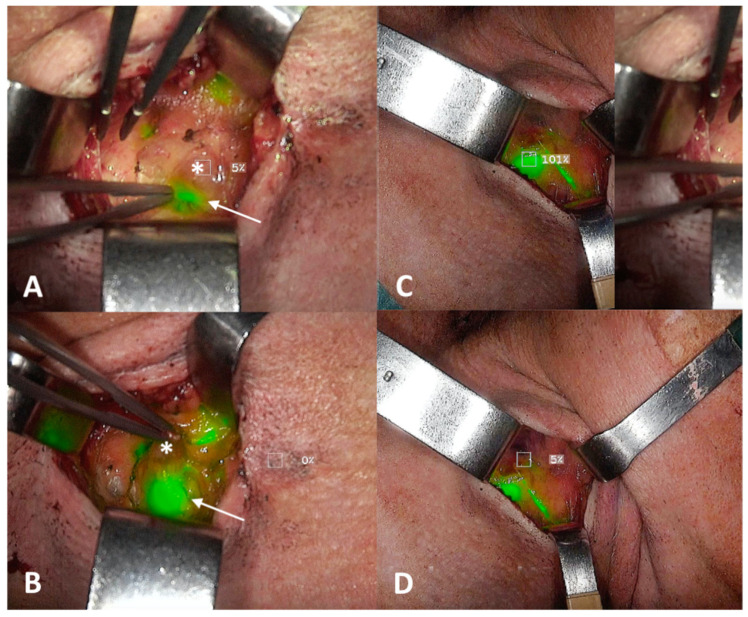
(**A**,**B**) Intraoperative identification with infrared camera at level III on the right side of a “hot” and “fluorescent” sentinel lymph node (SLN; white arrow) beneath a parasentinel node (asterisk): (**C**,**D**) Fluorescence was evaluated point by point by software estimation of the relative indocyanine green (ICG) dye uptake of the putative SLN ((**C**), 101%) in comparison with adjacent lymphoid tissue ((**D**), 5%), defining a SLN-to-background ICG ratio; a percentage was provided in real time by moving the infrared camera all along the surgical field.

Transoral laser excision (TLE) with margin assessment was performed as initial step (type II glossectomy) [10], to reduce the expected “shine-through phenomenon” on subsequent nodal dissection. ICG was injected directly at the margins of the tumor bed with four peripheral injections (2 mL of an ICG-distillated water solution divided in the four injections, with 20 mg of ICG at a 2.0 mg/mL concentration) immediately after TLE (Figure 1); a 10-minute window was considered for ICG to drain to the SLN(s). In general, if multiple SLNs are expected at different neck levels, dissection of these nodal hotspots is begun immediately after TLE under portable gamma detection probe guidance alone to have the putative SLNs exposed when ICG arrives (anticipating its possible washout), as in our case. A high-resolution ICG video-monitoring system (Stryker SPY Pinpoint^®^; Kalamazoo, MI, USA) was used to visualize the fluorescent dye intraoperatively: it consisted of a monitor, dedicated software for SLN-to-background ICG ratio estimation and a high-resolution camera with a near-infrared fluorescence (NIRF) laser covered by a single-use sterile sleeve. The identification of a “hot” and “fluorescent” lymph node gave reassurance that SLN was properly found, avoiding unnecessary dissection of neighboring para-SLNs (Figure 3). An in vivo comparative assessment with adjacent lymphoid tissue (the so-called “background noise”) was also performed in terms of radioactivity (with the hand-held gamma detection probe) and retained fluorescence to achieve double control. Radioactivity level as assessed as counts per second (cps); a ratio of at least 1:10 distinguished SLN from background noise. Fluorescence was evaluated point by point by software estimation of the relative ICG dye uptake of the putative SLN in comparison with adjacent lymphoid tissue (SLN-to-background ICG ratio): a percentage was provided in real-time by moving the NIRF camera all along the surgical field (Figure 3). However, since no thresholds were validated in this specific field, to date, the judgement as to whether a lymph node should be considered “fluorescent” or not remains qualitative; indeed, as demonstrated in Figure 3, the retained fluorescence within SLN is very easily distinguishable by the absence of fluorescence in adjacent tissues, especially para-SLN. Radioactive and optical control were performed both in vivo and ex vivo to obtain further confirmation. At left IB nodal hotspot, no SLN was found intraoperatively; conversely, one SLN was identified and removed at left IIA, left III and right III nodal hotspots. A para-SLN was also excised at level III on the right side (Figure 3). Intraoperative nerve monitoring (NIM Vital^®^, Medtronic; Minneapolis, MN, USA) was employed for level IB dissection to aid preservation of the mandibular branch of the facial nerve.

**Figure 4:**
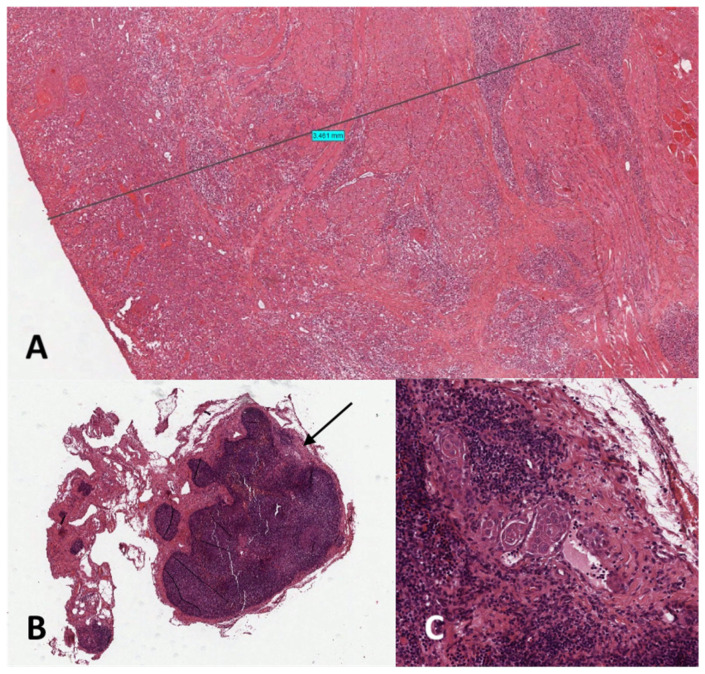
(**A**) A moderately differentiated squamous cell carcinoma (SCC) was proven in the glossectomy specimen with a depth-of-infiltration of 3.5 mm (pT1) H&E, magnification × 10. (**B**,**C**) A step serial sectioning protocol was applied for sentinel lymph node (SLN) histopathological analysis, founding a micrometastasis (diameter: 0.5 mm; black arrow, (**B**); highly magnified, H&E, magnification × 3. (**C**) in the left IIA SLN, H&E, magnification × 25.

Postoperative course was uneventful. At permanent sections, a moderately differentiated SCC was proven in the glossectomy specimen with a depth of infiltration of 3.5 mm (pT1) and a micrometastasis (diameter: 0.5 mm) in left IIA SLN (pN1sn(mi)) after regular step serial sectioning (Figure 4); other SLNs and para-SLNs were negative for further localization. Surgical margins were adequate. Given these circumstances, after consideration within our multidisciplinary tumor (MDT) board, a completion left modified radical neck dissection was performed 3 weeks later. In cases of positive SLNB, it is crucial to perform completion neck dissection (CND) within 3–4 weeks of the former procedure to avoid formation of extensive scar tissue between residual lymph nodes and neck neurovascular bundle, which would impair proper CND or increase morbidity, thus jeopardizing the benefits of the SLNB procedure. No other nodal metastases were found at definitive histology (1/36 N+ (mi)). Thus, no adjuvant therapies were advised. The patient is without evidence of disease at 38-month follow-up.

### 2.2. Case #2

**Figure 5:**
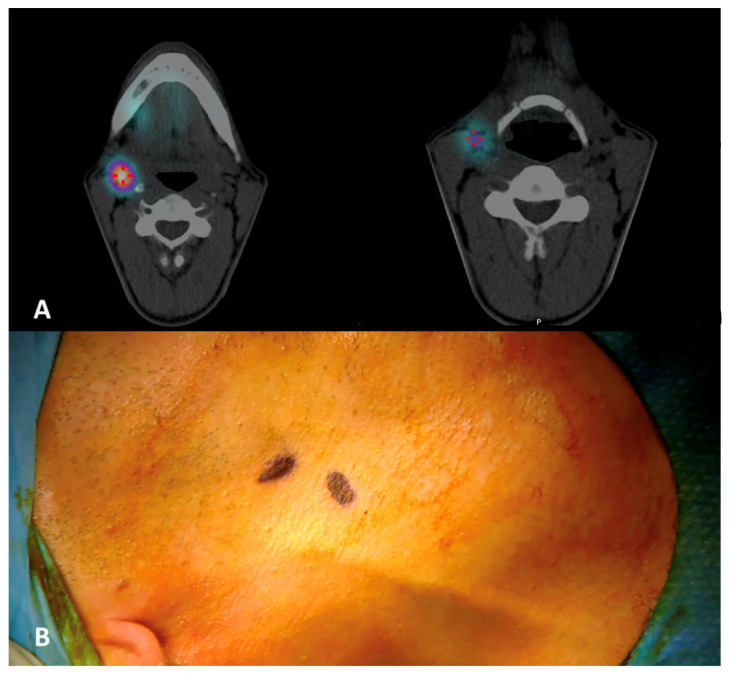
(**A**) Preoperative SPECT/CT with identification of two nodal hotspots (lighter circular areas encircled by a purplish ring) at levels IIA on the right side, one more cranially (**left**) and one more caudally (**right**). (**B**) Skin landmarking.

A 34-year-old man with a histologically proven right ventral tongue margin cT1 cN0 SCC was submitted to this protocol. The patient underwent the same preoperative workup, ruling out obvious neck metastases, and a lymphoscintigraphy with ^99m^Tc-Tilmanocept was performed about 4 h before surgery with four submucosal injections around the tumor (0.1 mL per injection, 0.4 mL of total dose, 17 MBq). On planar lymphoscintigraphy and SPECT/CT, two distinct nodal hotspots were identified at levels IIA on the right side: one more cranially at the level of the posterior belly of the digastric muscle, and another one more caudally just above the hyoid bone at the interface between jugulo-digastric and middle jugular nodes (Figure 5). Skin landmarking was performed at the end of procedure with the patient in a surgical position with the head hyperextended to aid subsequent planning. Transoral laser excision (TLE) was performed as initial step (type II glossectomy) [10], again to reduce the expected “shine-through phenomenon” of the injection site on the ventral tongue over the adjacent jugulo-digastric area. ICG was injected at the margins of the tumor bed with four peripheral injections immediately after TLE (2 mL of an ICG-distillated water solution divided in the four injections, with 20 mg of ICG at a 2.0 mg/mL concentration); as highlighted above, a 10-minute window was considered for ICG to drain passively to the specific SLN(s). The same high-resolution NIRF system as before was used (Stryker SPY Pinpoint^®^) to visualize ICG fluorescence intraoperatively and guide surgical dissection in real time.

**Figure 6:**
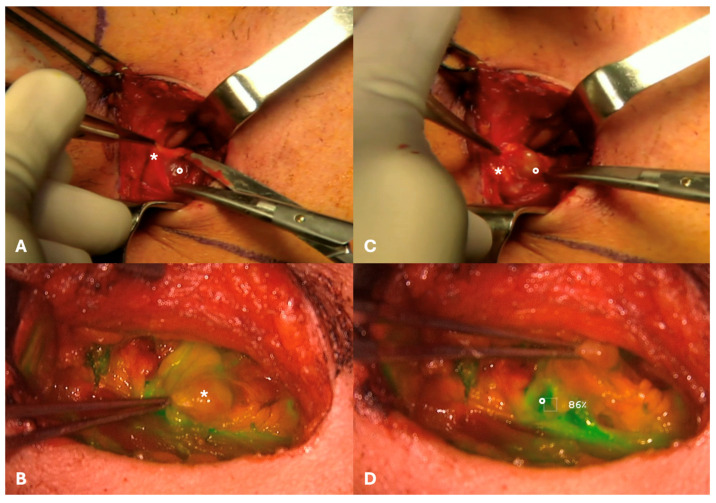
Intraoperative view during dissection within the right level IIA hotspot highlighting the exposure of a para-SLN (asterisk, grasped with a forcep; (**A**,**B**)) and an SLN emerging from deep within it (circle; (**C**,**D**)), with optical guidance allowing proper discrimination between them. In particular, fluorescence imaging allows for better spatial resolution than radioactive guidance because of the dimensions of the portable gamma detection probe.

The proper SLN was identified as “hot” and “fluorescent”, a double control that allowed fine discrimination between SLN and neighboring para-SLNs with better spatial resolution than radioactive guidance alone because of the dimensions of the portable gamma detection probe (Figure 6). An in vivo comparative assessment with the “background noise” was also performed in terms of radioactivity and retained fluorescence (SLN-to-background ICG ratio). Two SLNs were identified and removed at right level IIA hotspot (one more cranially, another one more caudally, as expected), together with 3 adjacent para-SLNs, which were consensually removed to avoid disrupting the capsule of the SLN while attempting to separate the lymph nodes.

**Figure 7:**
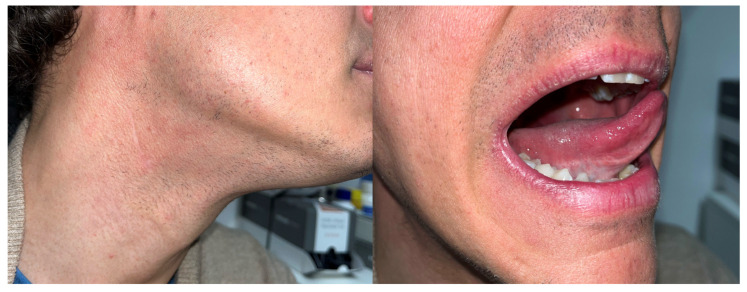
Postoperative findings at a 6-month follow-up both on the neck side (**left**) and on the tongue side (**right**), with excellent aesthetic and functional outcome.

Postoperative course was uneventful. At permanent sections, a well-differentiated SCC was proven in the glossectomy specimen with a depth of infiltration of 0.5 mm (pT1); SLNs and para-SLNs were negative for further localizations with regular step serial sectioning protocol after both hematoxylin–eosin and immunohistochemistry staining (using anti-pancytokeratin AE1AE3 antibody). Surgical margins were adequate. Given these circumstances, after consideration within our multidisciplinary tumor (MDT) board, no adjuvant therapies were advised. The patient is without evidence of disease at a 24-month follow-up (Figure 7).

**Figure 8:**
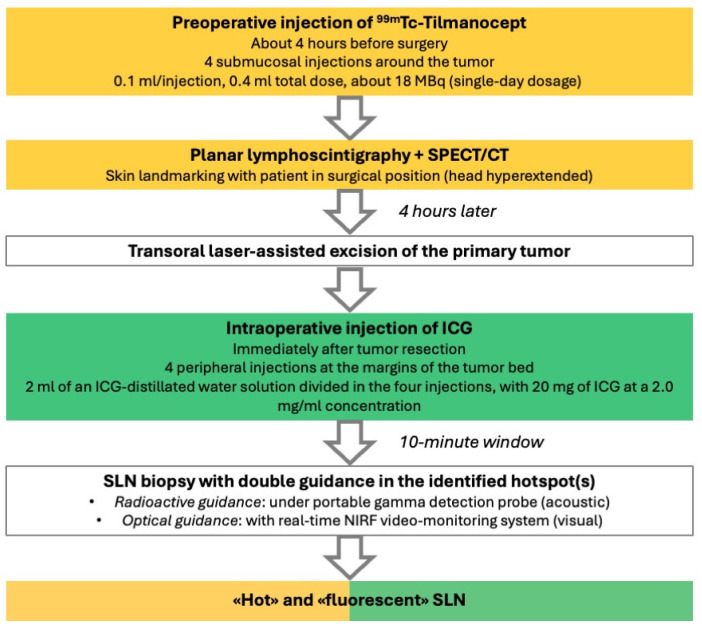
A stepwise description of the proposed multimodal protocol for sentinel lymph node biopsy with double guidance. SPECT/CT: single-photon emission computed tomography/computed tomography; ICG: indocyanine green; SLN: sentinel lymph node.

To date, four patients affected by early-stage OCSCC in shine-through areas (ventral tongue, FOM) have been enrolled for this multimodal protocol of SLNB; their main characteristic are listed in Table 1, whereas the steps in the proposed procedure are illustrated in Figure 8.

## 3. Discussion

SLNB is currently considered a viable alternative to END for the management of cN0 OCSCC, as widely reported [1,2,3]. Despite its oncological validity, some controversies remain, especially when dealing with FOM and ventral tongue tumors, the so-called “shine-through subsites”. Furthermore, until now, SLNB has failed to achieve wide adoption, probably for reasons related to the specific learning curve which this practice requires [2].

The “shine-through phenomenon” is indeed the main obstacle to SLNB sensitivity, as widely reported [2]. Systematic superselective level IB dissection has been considered, in many cases, as a possible solution: it provides the proper accuracy in identifying level IB hotspots, even when preoperative lymphoscintigraphy-SPECT/CT is negative at that level, especially within the “preglandular fat pad” [4,5]. However, this approach undoubtfully impairs the theoretical advantages of SLNB in terms of surgical time, skin incisions, and the risk posed to the mandibular nerve. Therefore, although effective, systematic superselective level IB dissection seems unreasonable as a definitive solution.

The regular introduction of both ^99m^Tc-Tilmanocept and optical guidance with ICG and the NIRF system provided further advancements in this field. For instance, in its multicenter validation trial, ^99m^Tc-Tilmanocept demonstrated a false negative rate of 0% in FOM tumors, probably because of its receptorial nature (it is a CD206 receptor-targeted radiotracer, which is retained in the reticuloendothelial cells of SLN) and small size (rapidly clearing the injection site) [6,7]. Nevertheless, den Toom et al. proved that the SLN-to-injection-site ratios were similar for both ^99m^Tc-Tilmanocept and the traditionally employed ^99m^Tc-nanocolloid because of the lower radioactive uptake in SLN for ^99m^Tc-Tilmanocept when compared to colloids (1.95% vs. 3.16%; *p* = 0.010), essentially underplaying the potential advantages of this new radiotracer per se [7]. Conversely, optical guidance with ICG as a fluorescent medium and a real-time NIRF system added a tremendous tool for providing intuitive intraoperative guidance, with a higher spatial resolution than traditional radioactive guidance (whose portable gamma detection probe is not especially useful in a very limited surgical field) [8]. However, ICG suffers from an unrestricted lymphatic migratory pattern (it drains quickly and passively to the SLN, with a rapid washout and, therefore, uptake in downstream non-SLN) and low tissue penetration power (1–2 cm), which makes its transcutaneous application unfeasible [8]. Coupling ICG to ^99m^Tc-nanocolloid through a non-covalent binding could represent a further refinement of the procedure. In the series by Christensen et al., [5] on 50 patients affected by tumors located in the anterior oral cavity, the false omission rate was 0%, and in 46% of the patients additional SLNs in level I were detected only with optical guidance. However, SLN in the “preglandular fat pad” was optically identified only during the superselective dissection of that level, since the hotspot was masked on preoperative lymphoscintigraphy and SPECT/CT, probably because of the non-receptorial nature of colloids [5]. 

The combination of ^99m^Tc-Tilmanocept with ICG in a single, hybrid tracer is not yet approved for clinical use by the FDA/EMA. Therefore, the proposed ICG fluorescence-guided SLNB protocol with ^99m^Tc-Tilmanocept used as a radiotracer seems a viable “surrogate”, which is designed to overcome these specific issues. In particular, a multimodal, stepwise approach is advised: after ^99m^Tc-Tilmanocept injection, preoperative SPECT/CT can identify the specific nodal hotspot(s) and, thus, define the surgical “roadmap”; subsequently, a portable gamma probe is used to intraoperatively pinpoint the SLN location within each nodal hotspot, with high specificity but limited spatial resolution. Finally, a NIRF system takes advantage of the fluorescence emitted by the ICG to provide intraoperative, high-definition optical guidance within each nodal hotspot with higher spatial resolution, allowing the safe identification of a “hot” and “fluorescent” SLN (i.e., double control). Effective optical guidance may indeed strengthen the radioguidance, increasing the sensitivity of the whole procedure, especially in terms of spatial resolution [11]. This is of paramount importance in the so-called “shine-through areas”, in which a substantial reduction in the unnecessary dissection of para-SLN nodes can be achieved. This latter consequence may further reduce morbidity, mostly in the submandibular region, and aid pathologists in step-serial analysis by limiting the specimens provided. As stated above, although hybrid tracers do exist (i.e., ICG-^99m^Tc-nanocolloid) and have been validated for this purpose [11], they still do not include Tilmanocept for radioguidance, which would be ideal for such a complex anatomical site as the anterior oral cavity. Indeed, the ability of Tilmanocept to identify hotspots, even at level IB, near the injection site, was also demonstrated in our small series (Figure 2), and in experienced hands, could help to avoid the systematic resort to the superselective dissection of “preglandular fat pad”, preserving the theoretical advantages of SLNB. Thus, the proposed multimodal protocol may mimic the use of a single, hybrid tracer with simultaneous optical and radioactive receptorial guidance.

Our protocol has a huge limitation of which we are fully aware. Since ICG flows quickly and passively from the injection site to the specific nodal basin without being retained within lymph nodes (as opposed to ^99m^Tc-Tilmanocept), it may wash out by the time the SLN is retrieved if it is injected at the beginning of the procedure (before TLE), as previously performed by Kågedal et al. [12], or even at the point of lymphoscintigraphy. Therefore, we decided to inject it directly at the margins of the tumor bed immediately after TLE; a 10-minute window was considered for the ICG to drain to the SLN(s), according to previous reports [1]. Hence, the correspondence in terms of SLN identification between Tilmanocept (injected preoperatively) and ICG (injected intraoperatively, after tumor resection) is questionable, and should be validated in properly powered studies of it is to be used on a wider scale. This is clearly a hypothesis-generating case series, and further assessments are needed to validate these findings. In particular, a within-patient comparison between the hotspot(s) defined by the radioactive guidance of ^99m^Tc-Tilmanocept (injected before surgery) and by the optical guidance of ICG (injected in the operative room after tumor resection) should be performed to eventually demonstrate the concordance/mismatch between the two methods, their accuracy in determining SLNs (in terms of sensitivity and specificity), the proper flow-through time of ICG outside SLNs (affecting the timing of ICG injection and SLN dissection) and the amount of unnecessary paraSLN dissected. This validation will probably need to be carried out in a multicenter setting to achieve the necessary statistical power. Currently, optical guidance in these settings should be intended only as an aid to radioactive-assisted dissection, which should remain the gold standard, especially if mismatches are encountered. Finally, we should underline that in our very small series, no mismatches were detected.

As demonstrated above, the ultimate refinement of the SLNB procedure will be the introduction of a single, hybrid tracer combining the aforementioned advantages. In preclinical models, this is already a reality: Guo et al. [13] coupled a fluorescent tag with similar properties to ICG (IRDye800 CW) with Tilmanocept through a covalent binding (with a lesser risk of uncoupling and flow-through of the fluorescent tag outside SLN than with non-covalent ICG-^99m^Tc-Nanocoll) and used it for SLNB of the oral cavity in rabbit models. There was a 98.0% agreement between the SLNs identified using fluorescence and those identified with radioactivity, with a Cohen’s kappa coefficient of 0.884, representing a highly accurate adjunct to SLNB for oral cavity cancer [13]. In particular, the adopted system provided a strong fluorescence signal, which was even visible through the skin, possibly allowing the performance of the SLNB procedure with optical guidance alone. Future perspectives are, thus, related to the possibility of also adopting such a dual-labeled tracer in clinical practice, given the extent of the advantages that such a technique could bring, not only in oral cavity cancer. For instance, a reliable hybrid tracer could help in head and neck skin cancer with parotid SLN in enhancing its identification within the glandular parenchyma due to the superior spatial resolution of optical guidance over radioactive guidance, reducing the risk of facial nerve injury. Moreover, the possibility of detecting this dual-labeled, hybrid tracer with the current NIRF systems should be explored, with the aim of considering its large-scale adoption. Finally, considering the economic burden that using such a system would entail in the short term, an analysis should be conducted to demonstrate the actual superiority of this tracer, not only from a theoretical standpoint, but also in terms of its accuracy in detecting SLNs compared to the use of a single radioactive tracer. An analysis of long-term cost-effectiveness with the endpoints of oncological non-inferiority and morbidity would also be valuable.

## 4. Conclusions

In conclusion, ICG fluorescence-guided SLNB with ^99m^Tc-Tilmanocept as the radiotracer is a safe and effective procedure for SLN identification in early-stage OCSCC, in which the great sensitivity provided by ^99m^Tc-Tilmanocept is enhanced by the greater spatial resolution of ICG fluorescence. This approach could be valuable for identifying nodes in critical subsites, such as the FOM and ventral tongue. In these locations, the presence of “shine-through radioactivity” could obscure eventual level IB/IIA nodal hotspots, leading to inaccurate staging of the neck and potentially affecting treatment decisions. Finally, the proposed multimodal protocol could play a role in accelerating the recognized learning curve of SLNB in such a complex anatomical area. This is clearly a hypothesis-generating case study and further assessments are needed to validate these findings, especially in terms of Tilmanocept–ICG correspondence within the nodal basin and eventual ICG thresholds for quantitative discrimination of SLNs from para-SLNs.

## Figures and Tables

**Table 1:** Clinical cases enrolled for the multimodal protocol of sentinel lymph node biopsy with ^99m^Tc-Tilmanocept and indocyanine green fluorescence imaging.

#	Age (Years)	Site	cT	eDOI (mm)	Nodal Hotspots	pT	pDOI (mm)	Nodes Removed	pN (sn)	ND	pN	FU
1	71	Ventral tongue	1	3.7	L IB, L IIA, L III, RIII	1	3.5	3 SLN 1 paraSLN	Micrometastasis (0.5 mm) in LIIA SLN	Yes	1 (1/36)	NED 38 months
2	34	Ventral tongue	1	3.0	R IIA	1	0.5	2 SLN 3 paraSLN	0	No	0	NED 24 months
3	54	FOM	1	3.0	L IB	1	0.5	1 SLN 1 paraSLN	0	No	0	NED 19 months
4	41	FOM	1	4.5	R IIA, L IB, L IIA	1	2.0	3 SLN 2 paraSLN	0	No	0	NED 17 months

FOM: floor of mouth; eDOI: radiologically estimated depth of infiltration; pDOI: pathological depth of infiltration; SLN: sentinel lymph node; ND: completion neck dissection; FU: status at follow-up; NED: no evidence of disease.

## Data Availability

The data presented in this study are available on request from the corresponding author due to privacy restrictions from our Institution.

## Supplementary Materials

The following supporting information can be downloaded at: https://zenodo.org/records/11410361?token=eyJhbGciOiJIUzUxMiJ9.eyJpZCI (accessed on 1 June 2024). Video S1: Lympho-angiography example protocol for sentinel lymph node seeking in early-stage oral cancer combining Tilmanocept and indocyanine-green.
